# Risk Stratification for Breast Cancer Patient by Simultaneous Learning of Molecular Subtype and Survival Outcome Using Genetic Algorithm-Based Gene Set Selection†

**DOI:** 10.3390/cancers14174120

**Published:** 2022-08-25

**Authors:** Bonil Koo, Dohoon Lee, Sangseon Lee, Inyoung Sung, Sun Kim, Sunho Lee

**Affiliations:** 1 Interdisciplinary Program in Bioinformatics, Seoul National University, Seoul 08826, Korea; 2 Bioinformatics Institute, Seoul National University, Seoul 08826, Korea; 3 BK21 FOUR Intelligence Computing, Seoul National University, Seoul 08826, Korea; 4 Institute of Computer Technology, Seoul National University, Seoul 08826, Korea; 5 Department of Computer Science and Engineering, Seoul National University, Seoul 08826, Korea; 6 Interdisciplinary Program in Artificial Intelligence, Seoul National University, Seoul 08826, Korea; 7 MOGAM Institute for Biomedical Research, Yongin-si 16924, Korea; 8 AIGENDRUG Co., Ltd., Seoul 08826, Korea

**Keywords:** patient stratification, molecular subtype, survival outcome, genetic algorithm, gene set selection

## Abstract

**Simple Summary:**

Patient stratification is clinically important because it allows us to understand the characteristics and establish treatment strategies for a group. Transcriptomic data play an important role in determining molecular subtypes and predicting survival. In the case of breast cancer, although the order of prognosis according to molecular subtypes is well known, there is heterogeneity even within a subtype. Therefore, patient stratification considering both molecular subtypes and survival outcomes is required. In this study, a methodology to handle this problem is presented. A genetic algorithm is used to select a set of genes, and a risk score is assigned to each patient using their expression level. According to the risk score, patients are ordered and stratified considering molecular subtypes and survival outcomes. Consequently, informative genes for patient stratification with respect to both aspects could be nominated, and the usefulness of the risk score was shown through comparison with other indicators.

**Abstract:**

Patient stratification is a clinically important task because it allows us to establish and develop efficient treatment strategies for particular groups of patients. Molecular subtypes have been successfully defined using transcriptomic profiles, and they are used effectively in clinical practice, e.g., PAM50 subtypes of breast cancer. Survival prediction contributed to understanding diseases and also identifying genes related to prognosis. It is desirable to stratify patients considering these two aspects simultaneously. However, there are no methods for patient stratification that consider molecular subtypes and survival outcomes at once. Here, we propose a methodology to deal with the problem. A genetic algorithm is used to select a gene set from transcriptome data, and their expression quantities are utilized to assign a risk score to each patient. The patients are ordered and stratified according to the score. A gene set was selected by our method on a breast cancer cohort (TCGA-BRCA), and we examined its clinical utility using an independent cohort (SCAN-B). In this experiment, our method was successful in stratifying patients with respect to both molecular subtype and survival outcome. We demonstrated that the orders of patients were consistent across repeated experiments, and prognostic genes were successfully nominated. Additionally, it was observed that the risk score can be used to evaluate the molecular aggressiveness of individual patients.

## 1. Introduction

Patient stratification is clinically important because distinct mechanisms of disease or specific responses to treatment can be determined [1]. It is clinically effective and useful to establish a specific treatment strategy for patients by analyzing the common biological mechanisms of the subgroup. Traditionally, patients were divided into subgroups based on the insights of clinicians. Advances in high-throughput sequencing technologies allow researchers to measure transcriptomic molecular profiles for individuals. This valuable transcriptomic information has made it possible to define molecular subtypes, especially in cancer studies, since about a decade ago [2,3,4,5]. In addition, genes associated with clinical outcome have been used to predict patient prognosis and revealed molecular mechanisms of disease [6]. These prognostic genes can also be detected using transcriptome data by survival analysis [7].

The definition of molecular subtypes using transcriptome data has been successful in clinical practice. The gene sets used to define molecular subtypes are usually derived by analysis of differentially expressed genes. Furthermore, sparse logistic regression (sparse LR) is widely used for gene set selection [8,9,10,11,12]. Usually, molecular subtypes of cancer have been determined by clustering methods using mRNA expression levels [13]. For example, Prediction analysis of microarray 50 (PAM50) subtypes [14] are widely used in the clinical setting to characterize breast cancer patients [15]. Another example is consensus molecular subtypes (CMSs) of colorectal cancer, which displayed well-established clinical and prognostic relationships with biological characteristics [16].

Survival prediction is one way to evaluate an individual’s prognosis. Prediction of survival outcome using transcriptome data has also been successful. For lung adenocarcinoma, gene expression signatures were successfully used to predict survival in a multi-institutional setting [17]. Prognosis-related genes are usually identified through survival analyses, and their combinations are used to predict the survival time of each sample. The Cox-model-based filter (Cox-filter) is an approach to finding prognostic signatures [18]. Recently, studies using multi-omics for optimal disease models are also being conducted. For example, Maui [19] is a method for representing multi-omics data as clinically relevant latent factors using a stacked variational autoencoder. Moreover, deep learning-based survival prediction methods are being actively studied [20,21,22].

Breast cancer is one of the most common types of cancer worldwide, with clinical studies of molecular subtypes and survival outcomes [23,24]. Usually, breast cancers are divided into four subtypes, luminal A (LumA), luminal B (LumB), her2-enriched (Her2), and basal-like (Basal) by expression of the immunohistochemistry (IHC) markers (Table 1), and the order of patient outcomes according to molecular subtypes is well-known [15,25]. Patients with luminal subtypes show a better prognosis and less aggressive characteristics. On the other hand, patients with the Basal subtype show worse prognosis and more aggressive characteristics such as a higher potential for metastasis. However, molecular subtype-based medicine has a limitation in that there is heterogeneity even within a subtype. For instance, some patients with LumA show a worse prognosis, and some patients with Basal show a better prognosis.

Nevertheless, there is no patient stratification method that considers molecular subtype and survival outcome simultaneously. The previous methods only focus on one of the two aspects. For instance, PAM50 defines subtypes of breast cancer, but it has limited power in predicting an individuals prognosis (Figure S1). The Stemness index is a useful measure for oncogenic dedifferentiation, and it showed a correlation between tumor pathology and clinical outcome [26]. Therefore, the stemness index can be used to predict survival outcomes, but it does not discriminate well-defined subtypes (Figure S2). Therefore, a technique for optimizing subtype information and survival outcome at the same time is required.

The goal of this study is to develop a methodology that stratifies patients, considering molecular subtype and survival outcome simultaneously. Our method calculates risk scores by considering the expression level of a gene set for a linear ordering of patients considering both perspectives. However, finding the optimal order is to explore N! space for *N* patients, which is infeasible even for a small number of patients. Ordering patients is undertaken using gene expression quantities; thus, the selection of genes for patient stratification adds much more complexity to the already huge search space. To handle the huge search space, we used a genetic algorithm (GA) [27], which selects an appropriate gene set from transcriptome data. From the gene set, the risk score was calculated for each patient, and the samples were ordered and stratified according to the risk score. We applied this methodology to breast cancer patients and evaluated the results in an independent cohort. In this experiment, we were able to stratify patients in terms of both molecular subtype and survival outcome. The result would be useful for clinical applications by combining molecular subtype-based medicine and survival-based prognosis prediction. In addition, since this methodology is based on a gene set, informative genes related to prognosis while distinguishing molecular subtypes were identified. Finally, it was observed that the risk score could be used to evaluate the molecular aggressiveness of individual patients.

## 2. Materials and Methods

**Figure 1:**
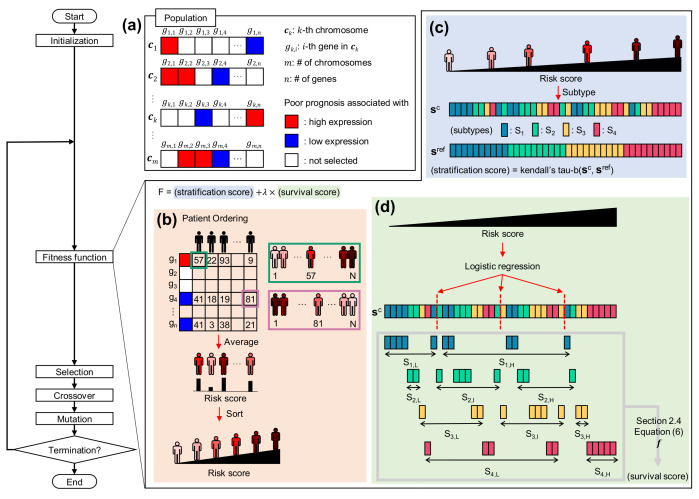
Overview of the genetic algorithm (GA) for patient ordering. (**a**) Chromosome representation. $c_{k}$ indicates the *k*-th chromosome in the population. $g_{k,i}$ indicates *i*-th genes in the chromosome. Red colored genes mean that the genes are selected to be related to worse prognosis when its expression is high. Blue colored genes imply that the genes are selected to be associated with a worse prognosis when their expression is low. White colored genes stand for unselected genes. (**b**) Patient ordering from a chromosome. Each patient is characterized by a risk score. Patients are ordered by each selected gene expressed as a ternary representation. For genes related to worse prognosis when its expression level is high, the lower the patient’s expression level, the higher the rank is given (green box). On the contrary, for genes associated with a worse prognosis when its expression quantity is low, the higher the patient’s expression level, the lower the rank is given (violet box). Then, the ranks are averaged for each patient, and it is considered a risk score. Finally, the patients are ordered by the score. (**c**) The stratification score is calculated as Kendall’s tau-b correlation between the vector of subtype corresponding to ordered patients ($s^{c}$), and the vector stratified completely according to subtype order ($s^{\mathrm{ref}}$). (**d**) The survival score is computed by analyzing the survival of the groups divided within each subtype. Groups within each subtype are defined through logistic regression with risk score as a variable.

In this section, we introduce a novel computational methodology for patient stratification to calculate patient risk for simultaneous learning of molecular subtypes and survival information. Figure 1 shows the overall methodology for calculating the risk score via GA utilizing transcriptome data for given patient samples. The key is to calculate patients’ risk score, taking into account gene expression patterns and gene combinations. Using the risk scores, patients are sorted and stratified. In addition, important genes associated with patient risk can be provided. In the following section, we describe the details of the GA for patient stratification, considering both molecular subtypes and survival outcomes, from chromosome representation to evolutionary process.

### 2.1. Breast Cancer Patient Data Collection

As for breast cancer data, two data sets were collected: The Cancer Genome Atlas-Breast invasive Carcinoma (TCGA-BRCA) [28] and Sweden Cancerome Analysis Network-Breast (SCAN-B) [29]. The RNA sequencing-based gene expression profiles of TCGA-BRCA were downloaded from UCSC Xena [30]. The subtype information corresponding to the samples in the gene expression data was obtained from the supplementary material from Berger et al. [31]. Clinical data, including overall survival information, were acquired from UCSC Xena and TCGA-CDR [32]. All data of SCAN-B are available from the NCBI Gene Expression Omnibus (GEO) under the accession number GSE96058. Gene expression levels in both data sets were measured by FPKM, and the chromosomes in GA were constructed with 23,550 genes, which were measured in both data sets. Among survival information, overall survival information in both data sets was used. The number of samples with subtype information is summarized in Table 2, and restricted mean survival time and the number of samples treated with therapy are summarized in Tables S1 and S2.

Based on the fact that the results of some previous biomarker studies are difficult to be reproduced in other data sets [33], external validation of results in other independent cohorts is very important [34]. This being so, we devised an experimental setting where a gene set was selected using TCGA-BRCA data, and the gene set was validated on independent SCAN-B data.

### 2.2. Chromosome Representation for Gene Combination

Given transcriptome data of patient samples, each patient can be expressed as a value from gene combinations. To select an informative gene set with respect to molecular subtypes and survival information, basically, a chromosome can be represented as a binary vector of genes (i.e., 0: not selected and 1: selected). However, since high or low expression of a gene can be associated with a poor prognosis, it is necessary to consider the quantity of the expression level. For example, high expression of Ki-67 is related to poor prognosis [35], and low expression of TP53 is associated with poor prognosis [36]. Thus, a novel chromosome representation was devised to select a gene set using a *ternary representation*.

(1) $$c_{k}=\left(g_{k,1},g_{k,2},\ldots ,g_{k,n}\right)$$

(2) $$g_{k,i}\in \left\{+1,0,-1\right\},$$

where $c_{k}$ indicates the *k*-th chromosome in the population, and $g_{k,i}$ indicates *i*-th gene in $c_{k}$. For $g_{k,i}$, when $g_{k,i}$ is +1, it means that the gene is selected as having an association with a worse prognosis when its expression is high. On the contrary, −1 implies that the gene is selected as having an association with a worse prognosis when its expression is low. When $g_{k,i}$ is 0, it stands for an unselected gene. In Figure 1a, +1 is expressed in red, −1 is expressed in blue, and 0 is expressed in white, and *n* is the number of genes in a data set.

### 2.3. Deriving Patient Risk Score from Chromosome

The risk score of patients is drawn from the configuration of the chromosome. For each selected gene in the chromosome, the patients are ranked by gene expression level according to the value encoded in the gene $g_{i}$. As shown by a green box in Figure 1b, if $g_{i}$ has a value of +1, the lower the expression in the patient, the higher the patient’s rank. On the other hand, if $g_{i}$ takes a value of −1, the higher the expression in the patient, the higher the patient’s rank. This case is indicated by a violet box in Figure 1b. Then, the ranks from the selected genes are averaged for each patient, and the average values are considered a risk score for patients. Finally, the patients are ordered in accordance with the assigned risk score.

### 2.4. Fitness Function for Evaluating Order of Patients

A fitness function *F* takes the order of patients as input which is derived from the risk scores of patients. It gives output as a value of how well the order reflects the subtypes and survival outcomes simultaneously. In order to achieve two objectives, the fitness function is composed of two terms: *stratification score* and *survival score*. The stratification score is to evaluate the order in terms of subtype. The survival score is a score to assess that the groups of patients are well ordered and stratified according to prognosis. Thus, the fitness function can be represented as follows.

(3) F=(stratificationscore)+λ×(survivalscore),

where λ is a coefficient to modulate the balance between the effects of the two scores on *F*. In practice, in this study, it was set to 0.5 by considering the scale of stratification score and survival score.

Stratification score is to evaluate whether the patient order reflects the molecular subtypes well (Figure 1c). Let the subtype order corresponding to the patient order be a vector, $s^{c}$, and $s^{\mathrm{ref}}$ is a vector containing subtype order when the patients are completely stratified according to the predefined subtype order. For example, if there are four subtypes and their order is $S_{1}<S_{2}<S_{3}<S_{4}$, $s^{\mathrm{ref}}$ is presented as follows.

(4) $$s^{\mathrm{ref}}=\left(\underset{N_{1}}{\underset{︸}{S_{1},\ldots ,S_{1}}},\underset{N_{2}}{\underset{︸}{S_{2},\ldots ,S_{2}}},\underset{N_{3}}{\underset{︸}{S_{3},\ldots ,S_{3}}},\underset{N_{4}}{\underset{︸}{S_{4},\ldots ,S_{4}}}\right),$$

where $N_{k}$ indicates the number of patients with subtype $S_{k}$ in the data set. For breast cancer, LumA, LumB, Her2, and Basal correspond to $S_{1}$, $S_{2}$, $S_{3}$, and $S_{4}$, respectively. This is from the fact that the order of patient outcomes according to breast cancer molecular subtypes is well-defined [15,25]. Then, the stratification score is computed as Kendall’s tau-b coefficient [37] between $s^{c}$ and $s^{\mathrm{ref}}$ since Kendall’s tau-b statistic makes adjustments for ties.

(5) $$\left(\mathrm{stratification}\;\mathrm{score}\right)=\frac{P-Q}{\sqrt{\left(P+Q+T)\times (P+Q+U\right)}}$$

*P* is the number of concordant pairs, *Q* is the number of discordant pairs, *T* is the number of ties only in $s^{c}$, and *U* is the number of ties only in $s^{\mathrm{ref}}$. If a tied pair arises in both $s^{c}$ and $s^{\mathrm{ref}}$, it is not added to either *T* or *U*.

Next, the survival score is computed to evaluate whether the order of patients well divides patients with good prognosis and patients with poor prognosis within each subtype (Figure 1d). In order to divide patients into groups in each subtype, the boundaries are determined by logistic regression. The risk score is used as a feature, and the probability value for the boundary was set to 0.5. Since the boundaries are determined in one-dimensional space, subtypes at both ends are divided into two groups, and the others are divided into three groups. If there are four subtypes as in the previous example, $S_{1}$ and $S_{4}$ are split into two groups (($S_{1,L}$ and $S_{1,H}$) and ($S_{4,L}$ and $S_{4,H}$)), respectively, and $S_{2}$ and $S_{3}$ are divided into three groups (($S_{2,L}$, $S_{2,I}$ and $S_{2,H}$) and ($S_{3,L}$, $S_{3,I}$ and $S_{3,H}$)), respectively. Here, subscript L, I, and H are to indicate the comparative risk within each subtype, and are abbreviations of Low, Intermediate, and High, respectively. Then, the survival score is computed as follows.

(6) $$\left(\mathrm{survival}\;\mathrm{score}\right)=\frac{1}{6}\left\{f\left(S_{1,L},S_{1,H}\right)+f\left(S_{2,L},S_{2,I}\right)+f\left(S_{2,I},S_{2,H}\right)+f\left(S_{3,L},S_{3,I}\right)+f\left(S_{3,I},S_{3,H}\right)+f\left(S_{4,L},S_{4,H}\right)\right\},$$

where *f* is a function to evaluate whether the order of two adjacent groups is correct and to determine whether their prognosis is different. The value of this function is calculated as the product of two values, as shown below.

(7) f(A,B)=c×l

The value of *c* is to judge whether the order of the two groups (*A* and *B*) is correctly arranged. The value of *l* is to determine whether the groups are significantly separated according to prognosis. *c* and *l* are computed through statistical methods, which are used in survival analysis, and their values are calculated as follows.

(8) $$c=\left\{\begin{array}{cc} 1 & \mathrm{if}\;\mathrm{HR}(B)>\mathrm{HR}(A)\\ -1 & \mathrm{otherwise} \end{array}\right.$$

(9) $$l=\left\{\begin{array}{cc} -\log_{10}\left(p\right) & \mathrm{if}\;p>b\\ -\log_{10}\left(b\right) & \mathrm{otherwise} \end{array}\right.,$$

where HR is the hazard ratio, which is the result of Cox’s proportional hazard model [38]. *p* is the *p*-value of the log-rank test result between *A* and *B*, and *b* is lower bound to ensure that all results are significant without focusing on a few parts. In other words, if the *p*-value is less than or equal to *b*, the value is clipped and *b* is used. It was set to 0.01 in this study.

Last but not least, in the initial iterations, it is difficult to set the boundaries properly since the order of elements of $s^{c}$ is random. Therefore, λ is set to 0 at the start to focus on subtype stratification during a few iterations. When the proportion of chromosomes that exceeds a certain stratification score (e.g., 0.75) exceeds a certain proportion in the population (e.g., 0.95), the survival score starts being considered in the fitness function.

### 2.5. Biological Operators in GA

Biological operators, which are essential components in GA [27], were used to find gene combinations that are better suited to compute the risk scores of patients. After the fitness values are calculated for all chromosomes in the population, parent chromosomes are selected to find more suitable gene sets in the next generation. Elitism and tournament selection were used to compose mating pools, and uniform crossover and mutation give opportunities by changing genes on the chromosomes. The GA was terminated when there was no improvement in the best fitness value within the population for 10 iterations. In addition, the concept of subpopulation [39] was utilized to find gene sets efficiently. In this study, the number of subpopulations in the population and the number of chromosomes in each subpopulation were empirically determined to be 10 and 150, respectively.

### 2.6. Comparison with Existing Approaches

Since there is no patient stratification method that simultaneously optimizes molecular subtype and survival outcome, we compared our method with the PAM50 gene list [14], widely used gene set selection methods [8,18], and gene sets for prognostic predictors of breast cancer [40,41,42,43,44]. For sparse LR and Cox-filter, gene sets were extracted using TCGA-BRCA data. In order to compare whether the gene sets divide the prognosis well within each subtype, k-means clustering and the log-rank test were performed. Clustering was used for grouping because it was not possible to know simply whether the expression level of a gene was associated with a poor prognosis. For comparison with our method, LumA and Basal were each divided into two clusters, and LumB and Her2 were divided into three clusters each. Moreover, logistic regression with 5-fold cross-validation was performed with 100 different data splits for comparison to evaluate the usefulness of the gene sets in subtype classification. For clustering and classification, log2-transformed FPKM was used after being standardized for each gene in the training data.

## 3. Results

### 3.1. Patient Stratification Considering Molecular Subtype and Survival Outcome Simultaneously

#### 3.1.1. Our Method Stratified Patients Considering Simultaneously Molecular Subtypes and Survival Outcomes

**Figure 2:**
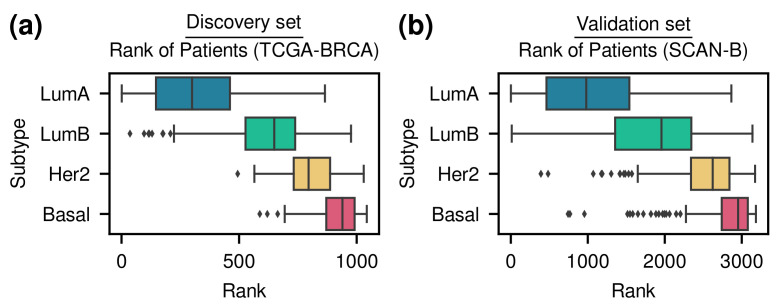
(**a**) TCGA-BRCA (discovery set). (**b**) SCAN-B (validation set). Ranking of patients determined by risk score. The lower the risk score, the higher the rank. Risk scores were computed from the 690 genes obtained from the genetic algorithm. The left edge of the box represents the first quartile (Q1), and the right edge represents the third quartile (Q3). The vertical line inside the box represents the median. Interquartile range (IQR) is defined as (Q3−Q1), and outliers are the samples outside 1.5 times the IQR above Q1 and below Q3. The whisker on the left goes from Q1 to the minimum, excluding outliers, and the whisker on the right goes from Q3 to the maximum, excluding outliers.

As a result of the GA, 690 genes were selected, and the patients in TCGA-BRCA data were stratified and ordered via the risk score with respect to both subtype and survival outcome. Among the 690 genes, 340 genes were selected as +1 value and 350 genes were selected as −1 value. By using this gene set, the order of patients in the SCAN-B data set was determined. Figure 2 shows the order of patients of TCGA-BRCA and SCAN-B data sets with respect to the molecular subtype. It was satisfactorily ranked in the order of the subtypes. In TCGA-BRCA data, the stratification score was 0.80 (correlation test *p*-value $=2.85\times 10^{-191}$), and in SCAN-B data, the stratification score was 0.68 (correlation test *p*-value $<10^{-277}$).

**Figure 3:**
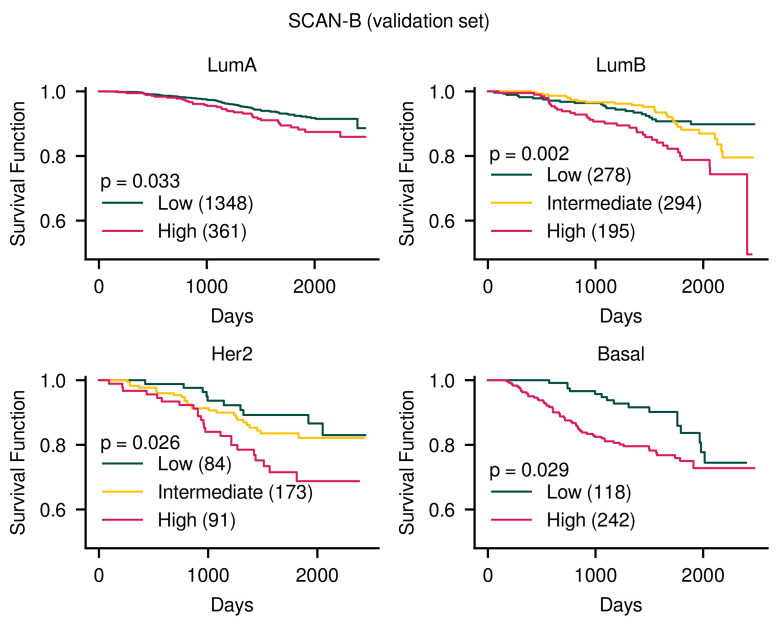
Kaplan–Meier curves for predicted risk groups within each subtype in SCAN-B data. Significant differences were observed among groups in the order of risk. In the TCGA-BRCA data set, the survival score was 2.00, and in the SCAN-B data set, the survival score was 1.22. The number in parentheses means the number of samples. *p*-values were results of multivariate log-rank tests. The results for TCGA-BRCA data are shown in Figure S3.

Furthermore, it showed significant results in the SCAN-B data set as well as the TCGA-BRCA data set when log-rank tests were performed among groups divided within each subtype (Figure 3 and Figure S3). Therefore, an appropriate order for each data set was obtained considering subtype and prognosis. In addition, significant results were observed even in the independent cohort.

#### 3.1.2. Previous Methods Do Not Consider Molecular Subtype and Survival Outcome Simultaneously

Existing patient stratification methods do not take account of molecular subtype and survival outcome at the same time. For example, even the well-established subtype of breast cancer, the PAM50 subtype, does not consider individual survival outcomes. Furthermore, as a result of clustering within each subtype using PAM50 genes, the gene set did not show significant results in dividing the group according to survival outcome (Figure S1). In other words, the PAM50 gene set is not suitable to distinguish between a group with a better prognosis and a group with a worse prognosis within each subtype. In addition, mRNAsi, which showed a correlation with tumor pathology and clinical outcome [26], is difficult to use for distinguishing LumB and Her2 (Figure S2a). It was also not suitable to show significant differences in survival outcomes within each subtype (Figure S2b). Moreover, although similar performance was shown in subtype classification when our methodology was compared with other gene set selection algorithms and gene sets for prognostic predictors of breast cancer, only our method showed significant results in prognostic stratification for all subtypes (Table 3).

### 3.2. Robustness of the Methodology for Constant Patient Ordering

In order to evaluate the stability of the methodology for constant ordering for patient stratification, 100 experiments were conducted with different random seeds. Then, Spearman’s rank correlation coefficients (SCCs) were computed for all pairwise combinations of patients’ order (Figure S4). As a result, the order of the patients was considerably constant. The median SCC for the TCGA-BRCA data set was 0.85 (correlation test *p*-value < $8.33\times 10^{-289}$), and the median SCC for the SCAN-B data set was 0.76 (correlation test *p*-value < $4.43\times 10^{-290}$). Thus, this methodology showed a robust result in finding an order that considers subtypes and survival simultaneously.

### 3.3. Usefulness of Fitness Function

**Figure 4:**
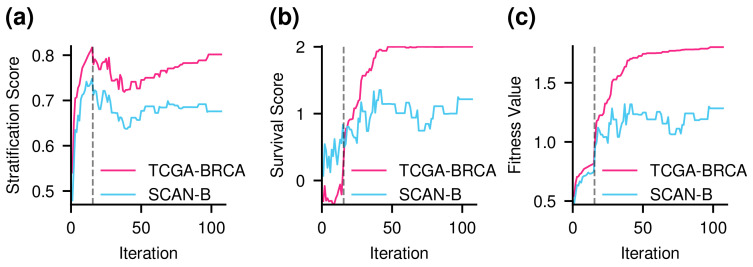
Scores of the best chromosome in each iteration. TCGA-BRCA is a discovery data set, and SCAN-B is a validation data set. The black dashed line stands for the point at which the survival score started to be calculated. (**a**) The stratification score tended to increase considerably before the survival score was considered. As the survival score started to be calculated, the stratification score decreased. As the survival score was saturated, the stratification score increased again. (**b**) The survival score increased significantly when it started to be considered in the fitness value. Although the survival score was not initially used to evaluate the order of patients, the values were computed and displayed. (**c**) As the population evolved, the fitness value increased not only in the discovery data but also in the validation data.

Thus far, we have seen that the fitness function learned subtype information and survival information well. Meanwhile, it is necessary to investigate how GA learns subtle relationships between subtype information and survival outcomes. It can be demonstrated by the change of the stratification score and the survival score according to iteration (Figure 4). The survival score was calculated from the 15th iteration. Before that, the stratification score increased considerably. After that, as the survival score started to be considered, the survival score increased fairly, and the stratification score showed a tendency to decrease. As the survival score began to saturate, the stratification score showed a tendency to increase again. Consequently, after the subtype order was arranged, the prognostic order was well learned, and when the prognostic order was established reasonably, the subtype order was well learned again.

Next, we conducted an experiment where only molecular subtypes were considered by setting λ to 0 (Figure S5). As a result, the order of patients was well arranged according to subtype. However, the order did not reflect the prognosis of the patients. In addition, the change of each score according to the λ value was observed (Figure S6). As a result, as the λ value increased, there was a tendency to further increase the survival score.

### 3.4. Frequently Selected Genes

**Figure 5:**
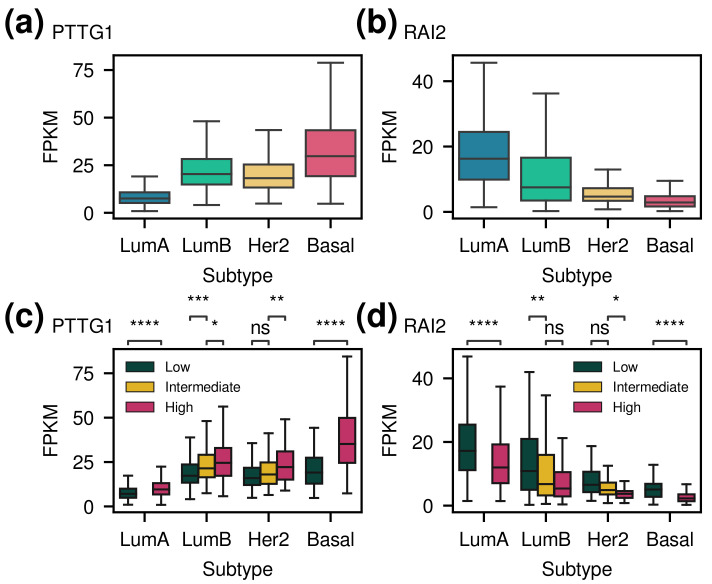
The gene expression levels of frequently selected genes in SCAN-B data (validation data). (**a**) Gene expression levels of *PTTG1* for each subtype. (**b**) Gene expression levels of *RAI2* for each subtype. (**c**) Gene expression levels of *PTTG1* for the risk groups predicted within each subtype. *PTTG1* was selected as a gene related to poor prognosis when its expression level is high. (**d**) Gene expression levels of *RAI2* for the risk groups predicted within each subtype. *RAI2* was selected as a gene associated with poor prognosis when its expression is low. The *p*-values are the results of the *t*-test with Bonferroni correction. Since all pairwise comparisons were significant (adjusted *p*-value <0.05) in (**a**,**b**), the significant levels were omitted. Outliers were omitted, and the plots for other frequently selected genes are shown in Figure S7. (ns: non-significant, *: p<0.05, **: p<0.01, ***: p<0.001, ****: p<0.0001).

The most selected genes among many genes would be important for distinguishing subtypes and associated with survival outcomes. Frequently selected genes showed significant differences in expression levels between subtypes or in groups within each subtype according to the predicted risk (Figure 5 and Figure S7). In addition, when the samples were divided into two groups based on average expression levels, significant differences were observed between the two groups (Figure S8).

Among the genes related to worse prognosis with high expression, *PTTG1*, *CENPL*, *CCNB2*, *FBXO5*, *UBE2C*, and *UBE2T* were selected more than 10 times out of the 100 experiments. *PTTG1*, *UBE2C*, and *UBE2T* are also included in the PAM50 gene list. *PTTG1* is well known for promoting the growth of breast cancer [45,46,47]. Moreover, *CENPL* was detected as one of the novel hub genes and served as a prognostic marker candidate in breast cancer [48], and high expression of *CCNB2* in breast carcinoma showed an association with disadvantageous clinical outcomes [49]. Furthermore, it was demonstrated that a higher expression level of *FBXO5* was significantly associated with a worse prognosis in breast cancer patients [50]. In addition, overexpression of *UBE2C* and *UBE2T*, which are ubiquitin conjugating enzymes, are known for promoting cell proliferation in breast cancer [51,52,53].

Among the genes related to worse prognosis with low expression, *LINC00160*, *RAI2*, *PVRL2* (*NECTIN2*), *PRKAG2-AS1*, and *MAPT-AS1* were selected more than 10 times out of the 100 experiments. *LINC00160* was served as a putative biomarker for ER-positive breast cancers by epigenetic analysis [54] and indicated prognostic significance in connection with the survival of breast cancer patients [55]. In addition, low expression of *RAI2* was reported as a poor prognostic marker in breast and colorectal cancer [56,57]. In hepatocellular carcinoma, low expression of *PVRL2* is associated with poor survival [58], and an antisense lncRNA *PPKAG2-AS1* inhibits malignant behaviors [59]. Furthermore, it was shown that an increased level of *MAPT-AS1* is related to better survival in breast cancer patients [60].

Among the genes related to poor prognosis with high expression, 168 genes were selected more than six times. Among the genes related to poor prognosis with low expression, 195 genes were selected. There was no intersection between the two gene sets. Additionally, gene set enrichment analyses were performed based on the biological process of gene ontology (GO) [61] using Enrichr [62], and *p*-values were adjusted for multiple comparisons by the Benjamini–Hochberg method. First, the analysis was carried out with 168 genes related to poor prognosis with high expression. As a result, 64 significant terms were enriched (adjusted *p*-value <0.05). The enriched terms were mainly related to the cell cycle. On the other hand, when the analysis was performed with 195 genes related to a worse prognosis with low expression, there were no significant terms.

In addition to using TCGA-BRCA as discovery and SCAN-B as validation, it would be useful to reverse the application to see if the gene set discovered by the SCAN-B data set is similar to the set of genes discovered by the TCGA-BRCA data set. Similar to when the TCGA-BRCA data set was used for discovery, 100 experiments were performed with different random seeds. The genes selected more than 10 times are listed in Table S5. *PTTG1* and *MAPT-AS1* were again nominated as frequently selected genes. Furthermore, *EXO1*, *KIF2C*, *MAPT*, *NAT1*, and *PTTG1* belonging to the PAM50 gene list were frequently selected. Among the frequently selected genes, it was reported that cancer cells are kept from oncogene-induced replication stress by overexpression of *CLSPN* [63]. Furthermore, *FOXM1* is a well-known transcription factor that is upregulated and overexpressed in aggressive phenotypes and has a poor prognosis in most human cancers as well as breast cancer [64,65,66].

**Figure 6:**
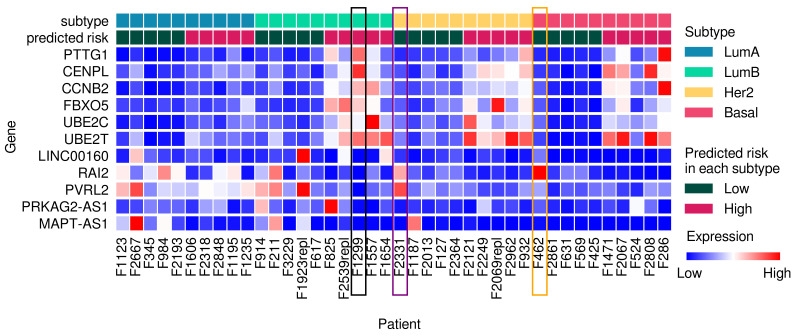
Expression patterns of frequently selected genes for the five patients with the lowest risk scores and the five patients with the highest risk scores within each subtype in SCAN-B data set. The amounts of gene expression were normalized for each gene within the entire data set. Among Basal patients, the *F462* patient had the smallest risk score and is highlighted with an orange box. *F462* had significantly high expression of the *RAI2* gene. Among Her2 patients, the *F2331* patient had the smallest risk score and is highlighted in a purple box. *F2331* had relatively high expression of the *RAI2* gene and *PVRL2* gene. *F1299* patient in the high-risk group of LumB is highlighted with a black box. F1299 showed high expression levels of genes that were related to worse prognosis with high expression and low expression levels of genes that were related to worse prognosis with low expression.

In Figure 6, the expression patterns of frequently selected genes for 40 samples are indicated. The samples were the five patients with the lowest risk scores and the five patients with the highest risk scores within each subtype. In each subtype, the five patients with the lowest risk scores had low expression levels of genes related to poor prognosis with high expression. Among the Basal patients, the patient *F462* with the lowest risk score (highlighted in an orange box in Figure 6) had considerably high expression levels of *RAI2*. Similarly, *F2331*, which was a sample with the lowest risk score among the Her2 patients (highlighted in a purple box in Figure 6), showed a relatively high expression levels of *RAI2* and *PVRL2*. *F1299*, which belonged to a high risk group in LumB (highlighted in a black box in Figure 6), showed high expression levels of genes, which were related to worse prognosis with high expression, and low expression levels of genes, which were related to worse prognosis with low expression. Although it is difficult to interpret a patient’s prognosis as the effect of a single gene, the results suggest that our methodology is useful for nominating novel marker genes for disease subtyping and survival modeling.

### 3.5. Comparison of Risk Score with Other Indices

**Figure 7:**
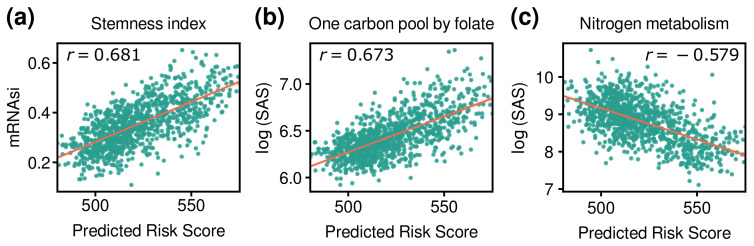
Scatter plots represent the relationship between the risk score and other indices. (**a**) The stemness index is a value to measure oncogenic dedifferentiation. The risk score showed a strong positive correlation with the mRNA stemness index ($p=9.62\times 10^{-140}$). (**b**) SAS is a score to measure the degree of activity of a subsystem (e.g., pathway). The risk score showed a strong positive correlation with one carbon pool by folate pathway (hsa00670) ($p=1.56\times 10^{-135}$). (**c**) The risk score showed a strong negative correlation with nitrogen metabolism (hsa00910) ($p=2.69\times 10^{-92}$). *r* indicates Pearson’s correlation coefficient, and *p*-values are the results of two-tailed correlation tests.

The Stemness index is a value to measure oncogenic dedifferentiation and is increased in metastatic tumors [26]. It showed a significant correlation with the risk score calculated for each patient (Figure 7a). Therefore, the patient risk scores indirectly reflected metastatic potential.

Additionally, Pearson correlation coefficients between the risk score and subsystem activation scores (SASs) of KEGG pathways were calculated. SAS is a score for measuring the degree of activity of a subsystem such as a pathway for each sample [67]. Among the pathways, one carbon pool by folate (hsa00670) showed the greatest positive correlation with the risk score (r=0.673; Figure 7b). That is, samples with a high risk score show high activity of this pathway. One carbon pool by folate pathway is one of the pathways known to be reprogrammed in cancer as a prognostic canonical pathway [68]. On the other hand, nitrogen metabolism (hsa00910) showed a high negative correlation with risk score (r=−0.579; Figure 7c). Nitrogen metabolism is related to cancer cell growth and proliferation [69], and it is controlled by glutamine [70,71,72]. *GLUD1* is included in the pathway, and its low expression is positively correlated with the activity of nitrogen metabolism and poor prognosis of breast cancer (Figure S9) [73,74].

## 4. Discussion

In this study, we proposed a novel computational methodology to stratify patient samples using transcriptome data, taking into account molecular subtypes and survival outcome simultaneously. Molecular subtype-based medicine has a limitation in that there is heterogeneity even within a subtype. For instance, some patients with LumA subtype have higher potentials of metastasis, while some patients with aggressive Basal subtype have lower metastasis potentials. Our methodology could overcome this limitation and would be useful for clinical practice by combining well-established molecular subtype-based medicine and survival-based prediction. The risk scores of patients considering both aspects can help establish diagnosis and treatment strategies for precision medicine. When a new patient visits a medical institution, it can help clinicians make decisions at the individual level.

Most of the previous studies that used GA for gene set selection formulated genes using binary representation in chromosomes [75,76,77]. We devised a ternary representation to consider not only selection but also the direction of association for prognosis. Thus, it allowed to automatically determine the direction and increase the interpretability of the results without post-processing. Furthermore, the proposed method can be used to determine genes related to other clinical information as well. For example, age, cancer grade and stage are also important features in evaluating a patient’s condition and establishing treatment plans. Moreover, if these clinically important variables are available together, more precise patient stratification will be possible.

There are some limitations in GA. Since better solutions are only compared to other solutions, the stop criterion is unclear. Furthermore, GA tends to converge to local optima or arbitrary points. These limitations can lead to inconsistent results and the selection of false positives. However, by performing GA several times and analyzing frequently selected genes, we were able to nominate useful novel genes for modeling subtypes or survival. For example, *RAI2*, which does not belong to the PAM50 gene list, was selected in the analysis, and it could play a role in helping to classify molecular subtypes of breast cancer.

We applied our methodology to breast cancer in this study. It was possible because the order of prognosis according to molecular subtype is well-defined for breast cancer [15,25]. However, this method can also be applied in other diseases where subtype ordering is possible. For instance, in colorectal cancer, it is known that the survival outcome is poorer in the order of CMS2, CMS3, CMS1, and CMS4 [16]. Moreover, there are some parts of our methodology that can be further modified or extended. For Cox’s proportional hazard model, which was used to compute survival score, we did not control other covariates that might affect prognosis, including age or stage at diagnosis. Additionally, transcriptomic data-based, not IHC-based, subtype information was utilized for validation. These would potentially confound the ability to extract prognosis-related genes because they may induce a bias or disconnection with a clinical practice [78]. Therefore, there is room for improvement in the selection of prognostic genes by considering non-molecular covariates as additional variables. In addition, logistic regression can be replaced by other more accurate classification algorithms for determining boundaries among subtypes. Furthermore, other clinically important attributes of an ordinal data type, such as cancer grade, can be utilized in the replacement of subtype information. This can also be applied to other diseases for which there is no well-defined subtype order. In such a case, it would be possible to stratify the patients and find important genes considering both clinical features and survival outcome.

In summary, we addressed the challenge of patient stratification for simultaneous learning of molecular subtypes and survival outcomes. However, there are still limitations to the proposed method. When calculating the risk score, patients were ranked based on the expression level of each gene. Although the rank-based methods are more robust to outliers, platforms and batches, there may be a loss of quantity information by converting the expression quantity to a rank. Therefore, a method using the expression quantity of the gene itself or a method applying the differential privacy mechanism for individuals privacy [79] could be more effective for representing individual patients. Next, interactions among genes were not directly considered. Network-based methods can be more powerful than methods of analyzing individual genes independently. Accordingly, as a follow-up study, we plan to investigate a network-based patient stratification method that simultaneously considers molecular subtypes and survival outcomes.

## Figures and Tables

**Table 1:** Summary of the molecular subtypes according to immunohistochemistry assay in breast cancer. ER, PR, HER2, and Ki-67 mean estrogen receptor, progesterone receptor, human epidermal growth factor receptor 2, and marker of proliferation, respectively.

Subtype	ER and/or PR	HER2	Ki-67
Luminal A (LumA)	ER+ or PR+	HER2−	Ki-67−
Luminal B (LumB)	ER+ or PR+	any	Ki-67+
Her2-enriched (Her2)	ER− and PR−	HER2+	any
Basal-like (Basal)	ER− and PR−	HER2−	any

**Table 2:** The number of samples for each subtype of breast cancer data. Values in parentheses mean the proportion of each subtype in the data sets.

Subtype	TCGA-BRCA	SCAN-B
LumA	563 (53.98%)	1709 (53.67%)
LumB	206 (19.75%)	767 (24.09%)
Her2	82 (7.86%)	348 (10.93%)
Basal	192 (18.41%)	360 (11.31%)
Total	1043	3184

**Table 3:** Performance comparison on SCAN-B data set. For prognosis stratification, the *p*-value of the log-rank test result within each subtype is shown, and significant results are marked in bold (*p*-value <0.05). The results of the TCGA-BRCA data set are shown in Table S3.

	Log-Rank Test (*p*-Value)			
	LumA	LumB	Her2	Basal
**GA (Ours)**	**0.033**	**0.002**	**0.026**	**0.029**
PAM50 [14]	0.248	0.267	0.780	0.894
sparse LR [8]	0.166	0.802	0.803	0.571
Cox-filter [18]	0.330	0.823	**0.012**	0.196
EndoPredict [40]	0.120	0.159	0.171	**0.033**
GENE70 [41]	0.140	0.094	0.845	0.570
GENE76 [42]	0.082	0.061	0.995	0.414
GENIUS M1 [43]	0.452	**0.019**	**0.014**	0.285
GENIUS M2 [43]	0.515	0.371	0.253	0.063
GENIUS M3 [43]	0.050	0.544	0.529	0.788
GGI [44]	0.282	0.637	0.810	0.584

## Data Availability

The implementation of a genetic algorithm for patient stratification is available at https://github.com/BonilKoo/patient_stratification (accessed on 5 July 2022). The RNA sequencing-based gene expression profiles of TCGA-BRCA were downloaded from UCSC Xena (https://xenabrowser.net/datapages/, accessed on 2 November 2021). The subtype information corresponding to the samples in the gene expression data was obtained from the supplementary material of Berger et al. [31]. Overall survival data were acquired from UCSC Xena and TCGA-CDR (https://gdc.cancer.gov/about-data/publications/PanCan-Clinical-2018, accessed on 2 November 2021). All data for SCAN-B are available from NCBI GEO under the accession number GSE96058 (ClinicalTrials.gov Identifier: NCT02306096).

## Supplementary Materials

The following supporting information can be downloaded at: https://www.mdpi.com/article/10.3390/cancers14174120/s1, Table S1: Clinical information from TCGA-BRCA dataset; Table S2: Clinical information from SCAN-B dataset; Table S3: Performance comparison on TCGA-BRCA data set (discovery data set); Table S4: Performance comparison on SCAN-B data set (validation data set); Table S5: Frequently selected genes when gene sets were discovered on the SCAN-B data set; Figure S1: Kaplan-Meier curves for clusters within each subtype; Figure S2: (a) mRNA stemness index (mRNAsi) for each subtype. (b) Kaplan-Meier curves for groups divided by mRNAsi; Figure S3: Kaplan-Meier curves for predicted risk groups within each subtype in TCGA-BRCA data; Figure S4: Spearman’s correlation coefficients between all pair-wise patient orders which come from 100 repetitive experiments; Figure S5: The results when only stratification score was considered (λ = 0). Figure S6: The scores according to the change in λ value. Figure S7: The gene expression levels of frequently selected genes for each subtype and the risk groups predicted within each subtype in SCAN-B data; Figure S8: Kaplan-Meier curves of frequently selected genes for SCAN-B data; Figure S9: The expression level of *GLUD1* showed a negative correlation with the risk score and a positive correlation with the activity of nitrogen metabolism.

## Abbreviations

The following abbreviations are used in this manuscript:

Basal	Basal-like
BRCA	Breast Invasive Carcinoma
*CCNB2*	Cyclin B2
*CENPL*	Centromere Protein L
*CLSPN*	Claspin
CMS	Consensus Molecular Subtype
Cox-filter	Cox-model based filter
ER	Estrogen receptor
*EXO1*	Exonuclease 1
*FBXO5*	F-Box Protein 5
*FOXM1*	Forkhead Box M1
FPKM	Fragments Per Kilobase of transcript per Million mapped reads
GA	Genetic Algorithm
GEO	Gene Expression Omnibus
*GLUD1*	Glutamate Dehydrogenase 1
GO	Gene Ontology
${}_{H}$	High
Her2	Human epidermal growth factor receptor 2
HR	Hazard Ratio
${}_{I}$	Intermediate
IHC	Immunohistochemistry
KEGG	Kyoto Encyclopedia of Genes and Genomes
*KIF2C*	Kinesin Family Member 2C
${}_{L}$	Low
*LINC00160*	Long Intergenic Non-Protein Coding RNA 160
LumA	Luminal A
LumB	Luminal B
lncRNA	long non-coding RNA
*MAPT*	Microtubule Associated Protein Tau
*MAPT-AS1*	MAPT Antisense RNA 1
mRNA	messenger RNA
mRNAsi	mRNA stemness index
*NAT1*	N-Acetyltransferase 1
NCBI	National Center for Biotechnology Information
PAM50	Prediction Analysis of Microarray 50
PR	Progesterone receptor
*PRKAG2*	Protein Kinase AMP-Activated Non-Catalytic Subunit Gamma 2
*PRKAG2-AS1*	PRKAG2 Antisense RNA 1
*RAI2*	Retinoic Acid Induced 2
RNA	Ribonucleic acid
*PTTG1*	Pituitary Tumor Transforming Gene 1
*PVRL2*	Poliovirus Receptor-related 2
SAS	Subsystem Activation Score
SCAN-B	Sweden Cancerome Analysis Network - Breast
SCC	Spearman’s Correlation Coefficient
sparse LR	sparse Logistic Regression
TCGA	The Cancer Genome Atlas
*TP53*	Tumor Protein P53
*UBE2C*	Ubiquitin Conjugating Enzyme E2 C
*UBE2T*	Ubiquitin Conjugating Enzyme E2 T
