# Supplementary material for: Risk Stratification for Breast Cancer Patient by Simultaneous Learning of Molecular Subtype and Survival Outcome Using Genetic Algorithm-Based Gene Set Selection
Source: Cancers (Basel). 2022 Aug 25;14(17):4120. doi: 10.3390/cancers14174120 (PMC9454699; doi:10.3390/cancers14174120)
Supplement: Supplementary file 1 [file cancers-14-04120-s001.zip › cancers-1826554-supplementary.pdf]

Table S1: Clinical information from TCGA-BRCA dataset. Restricted mean survival time was calculated as a period of up to five years. The number of samples treated with radiation for each subtype is shown. NaN indicates that the information is not available. Values in parentheses mean the proportion within each subtype.

Subtype	TCGA-BRCA			
	Restricted mean survival time (days)	Radiation therapy		
		YES	NO	NaN
LumA	1,725	285 (50.62%)	222 (39,43%)	56 (9.95%)
LumB	1,656	102 (49.51%)	81 (39.32%)	23 (11.17%)
Her2	1,525	35 (42.68%)	33 (40.24%)	14 (17.07%)
Basal	1,629	99 (51.56%)	71 (36.98%)	22 (11.46%)
Total	1,679	521 (49.95%)	407 (39.02%)	115 (11.03%)

Table S2: Clinical information from SCAN-B dataset. Restricted mean survival time was calculated as a period of up to five years. The number of samples treated with endocrine therapy and chemotherapy for each subtype is shown. NaN indicates that the information is not available. Values in parentheses mean the proportion within each subtype.

Subtype	SCAN-B						
	Restricted mean survival time (days)	Endocrine therapy treated			Chemotherapy treated		
		YES	NO	NaN	YES	NO	NaN
LumA	3,396	1,485 (86.89%)	215 (12.58%)	9 (0.53%)	391 (22.88%)	1,309 (76.59%)	9 (0.53%)
LumB	3,176	730 (95.18%)	35 (4.56%)	2 (0.26%)	369 (48.11%)	396 (51.63%)	2 (0.26%)
Her2	3,108	185 (53.16%)	160 (45.977%)	3 (0.86%)	243 (69.83%)	102 (29.31%)	3 (0.86%)
Basal	2,918	52 (14.44%)	302 (83.89%)	6 (1.67%)	273 (75.83%)	81 (22.5%)	6 (1.67%)
Total	3,266	2,452 (77.01%)	712 (22.36%)	20 (0.63%)	1,276 (40.08%)	1,888 (59.30%)	20 (0.63%)

Table S3: Performance comparison on TCGA-BRCA data set (discovery data set). Macro-averaged F1 score indicates unweighted mean of F1 score calculated for each subtype, and weighted-averaged F1 score indicates weighted mean of F1 score calculated for each subtype. For subtype classification, mean with standard deviation are shown. For prognosis stratification p-value of log-rank test result within each subtype is shown, and significant results are marked in bold (p-value < 0.05). The upward arrow in parentheses next to subtype classification task indicates that the higher the performance metric, the better. The downward arrow in parentheses next to prognosis stratification task indicates that the lower the p-value, the better. Since sparse LR was used to select a gene set on the TCGA-BRCA data set, sparse LR was not performed on the subtype classification task.

	Subtype classification (↑)			Prognosis stratification (↓)			
				Log-rank test (p-value)			
	Accuracy	Macro-averaged F1 score	Weighted-averaged F1 score	LumA	LumB	Her2	Basal
**GA (Ours)**	0.850 ± 0.022	0.810 ± 0.030	0.850 ± 0.022	**0.001**	$\mathbf{< 10^{-6}}$	$\mathbf{10^{-4}}$	**0.006**
PAM50	0.844 ± 0.058	0.827 ± 0.044	0.849 ± 0.053	0.895	0.199	0.609	0.520
sparse LR	-	-	-	0.090	0.733	**0.028**	0.480
mRNAsi	-	-	-	0.382	0.446	0.416	0.783
Cox-filter	0.870 ± 0.021	0.831 ± 0.030	0.869 ± 0.021	0.200	0.475	0.236	0.800
EndoPredict	0.754 ± 0.037	0.625 ± 0.063	0.739 ± 0.042	**0.016**	0.844	0.350	0.734
GENE70	0.796 ± 0.027	0.722 ± 0.037	0.799 ± 0.026	0.965	0.566	0.571	0.647
GENE76	0.770 ± 0.036	0.709 ± 0.040	0.778 ± 0.033	0.697	0.938	0.458	0.061
GENIUS M1	0.783 ± 0.032	0.722 ± 0.038	0.786 ± 0.029	0.237	0.191	0.542	0.192
GENIUS M2	0.683 ± 0.064	0.561 ± 0.065	0.670 ± 0.058	1	0.237	0.173	0.196
GENIUS M3	0.852 ± 0.022	0.804 ± 0.033	0.852 ± 0.022	0.184	0.207	0.678	0.648
GGI	0.816 ± 0.027	0.766 ± 0.034	0.815 ± 0.027	0.835	0.027	0.431	0.347

Table S4: Performance comparison on SCAN-B data set (validation data set). Macro-averaged F1 score indicates unweighted mean of F1 score calculated for each subtype, and weighted-averaged F1 score indicates weighted mean of F1 score calculated for each subtype. For subtype classification, mean with standard deviation are shown. The result on TCGA-BRCA data set (discovery data set) are shown in Table S1.

	Accuracy	Macro-averaged F1 score	Weighted-averaged F1 score
**GA (Ours)**	0.789 ± 0.014	0.765 ± 0.016	0.797 ± 0.013
PAM50	0.856 ± 0.013	0.822 ± 0.019	0.854 ± 0.014
sparse LR	0.819 ± 0.014	0.799 ± 0.016	0.823 ± 0.014
Cox-filter	0.746 ± 0.015	0.726 ± 0.017	0.753 ± 0.015
EndoPredict	0.600 ± 0.016	0.455 ± 0.020	0.607 ± 0.017
GENE70	0.757 ± 0.014	0.665 ± 0.021	0.754 ± 0.015
GENE76	0.736 ± 0.015	0.663 ± 0.019	0.739 ± 0.015
GENIUS M1	0.709 ± 0.017	0.673 ± 0.019	0.718 ± 0.016
GENIUS M2	0.562 ± 0.018	0.460 ± 0.021	0.575 ± 0.017
GENIUS M3	0.820 ± 0.015	0.781 ± 0.018	0.825 ± 0.014
GGI	0.769 ± 0.013	0.694 ± 0.018	0.764 ± 0.014

Table S5: Frequently selected genes when gene sets were discovered on the SCAN-B data set. Genes which are also frequently selected in the TCGA-BRCA data set are marked in bold. Genes belonging to the PAM50 gene list are underlined.

Genes associated with worse prognosis when expression is high	
Number of times a gene was selected	Genes
10	*AURKB*, *BUB1*, *CCNA2*, *CDC25A*, *CDCA7*, *CTSV*, *DEPDC1*, *EXO1*, *GTPBP2*, *MCUR1*, *ORC1*, *PARPBP*, ***PTTG1***, *RFC4*, *SGOL1-AS1*, *SKA3*
11	*BUB1B*, *CDT1*, *NCAPH*, *NEK2*, *POLQ*
12	*CENPA*, *CENPW*, *GTSE1*, *KIF2C*, *RAD51*, *TACC3*, *TTK*
13	*CLSPN*, *FOXM1*, *SPC24*

Genes associated with worse prognosis when expression is high	
Number of times a gene was selected	Genes
10	*ABAT*, *DNAAF1*, *FDXACB1*, *GRIA4*, *GRIK3*, *GRPR*, *IL17B*, *KDM4B*, *LINC00959*, *NEK10*, *NRIP1*, *RALGPS2*, *STARD10*, *SUSD3*, *TP63*
11	*F2RL2*, *FGD3*, *PTGER3*, *RAB30*, *RIMS4*
12	*LINC01016*, *NAT1*, *UBXN10*
13	*GSTM3*, *MAPT*, ***MAPT-AS1***, *TTC39A*
14	*TFF1*
17	*MAPT-IT1*

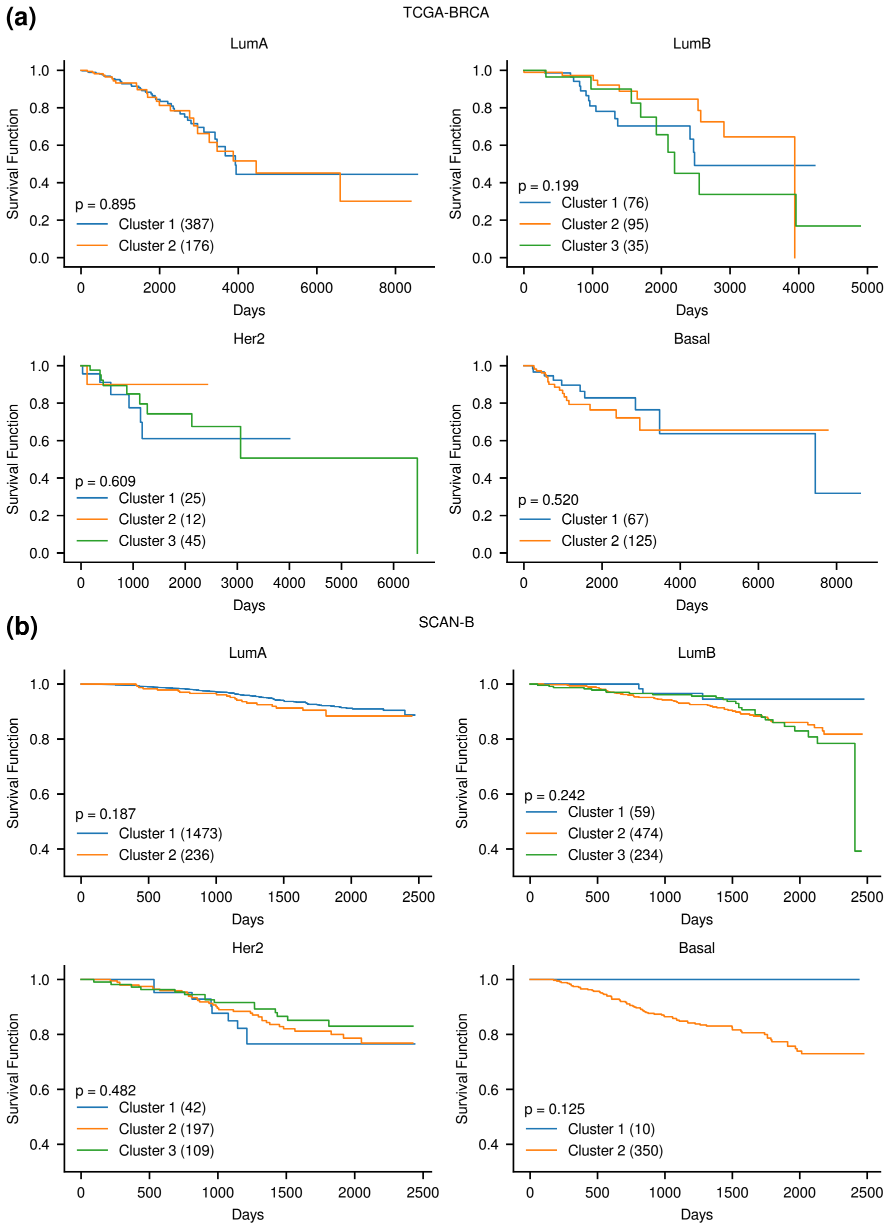

Figure S1: Kaplan-Meier curves for clusters within each subtype. K-means clustering was used to divide each subtype to subgroups using PAM50 genes. There were no significant differences in survival among the clusters in each subtype. P- values were results of multivariate log-rank tests. The number in parentheses means the number of samples. **(a)** TCGA-BRCA **(b)** SCAN-B

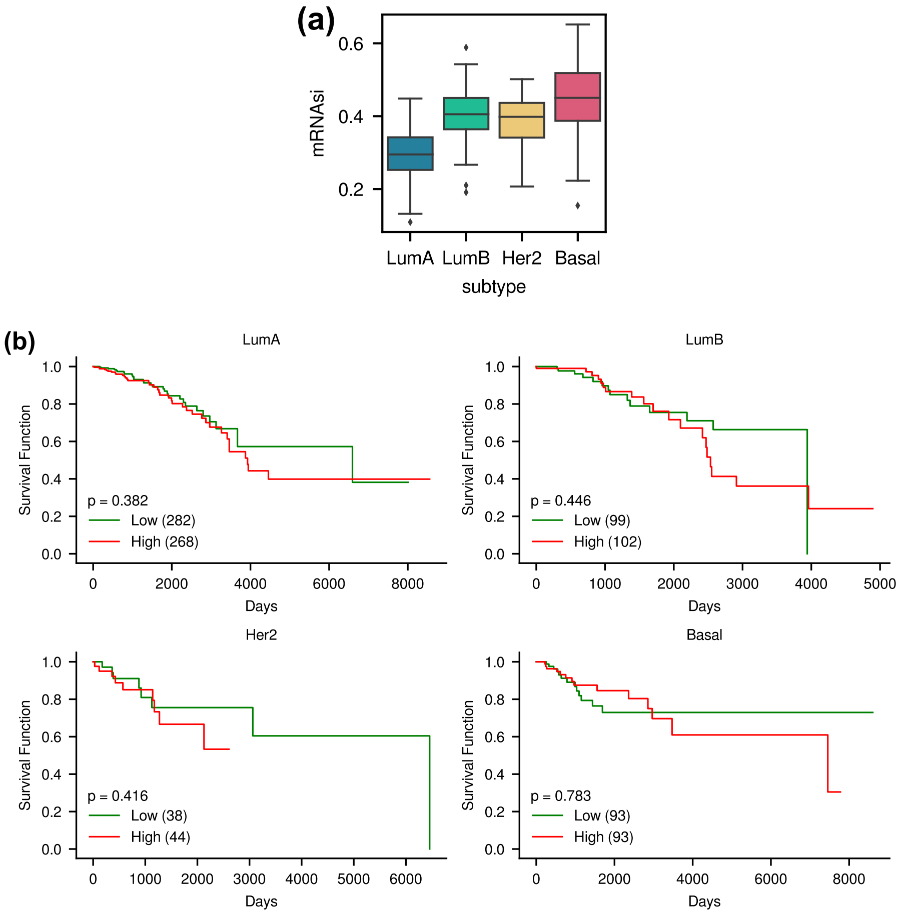

Figure S2: **(a)** mRNA stemness index (mRNAsi) for each subtype. When the samples were ordered by mRNAsi, the stratification score was 0.128. **(b)** Kaplan-Meier curves for groups divided by mRNAsi. The samples were split into two groups based on average mRNAsi values within each subtype. There were no significant differences in survival between groups. The number in parentheses means the number of samples. P-values were results of multivariate log-rank tests.

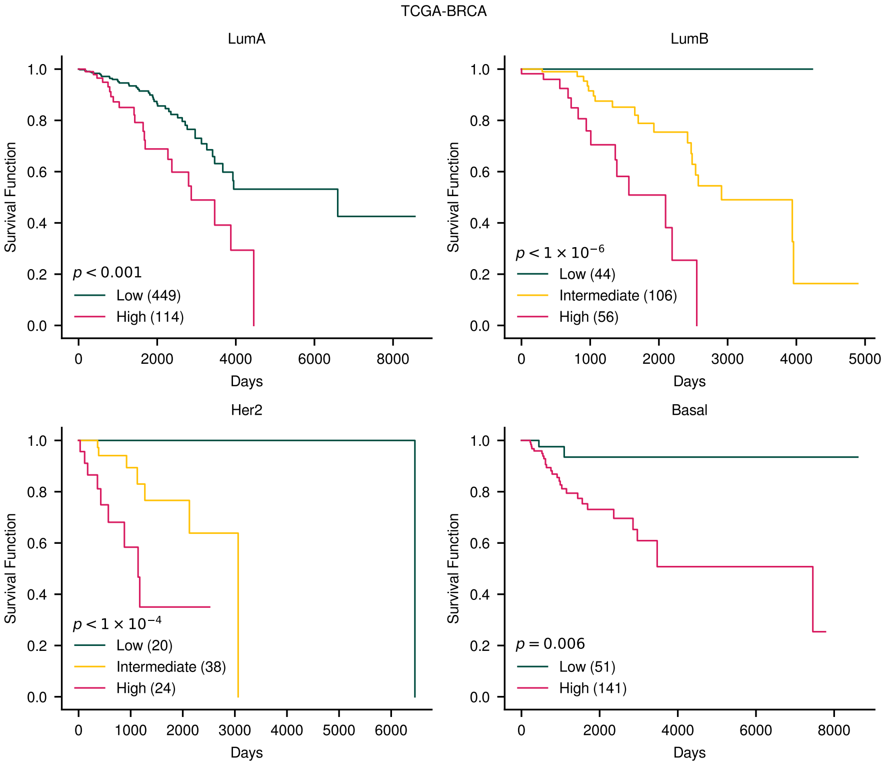

Figure S3: Kaplan-Meier curves for predicted risk groups within each subtype in TCGA-BRCA data. Significant differences were observed among groups in the order of risk. The number in parentheses means the number of samples. P-values were results of multivariate log-rank tests.

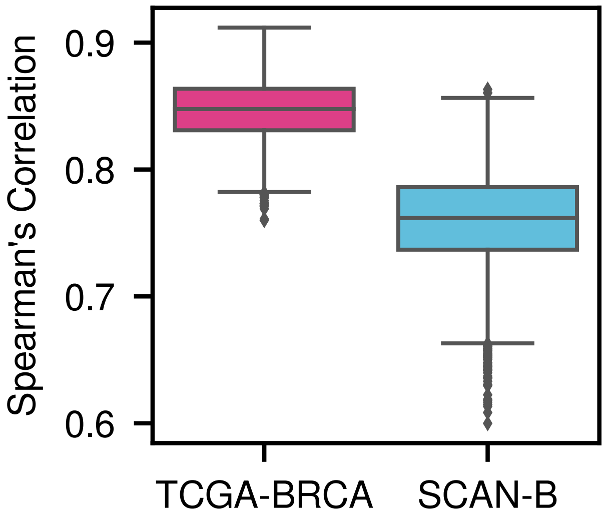

Figure S4: Spearman's correlation coefficients between all pair-wise patient orders which come from 100 repetitive experiments.

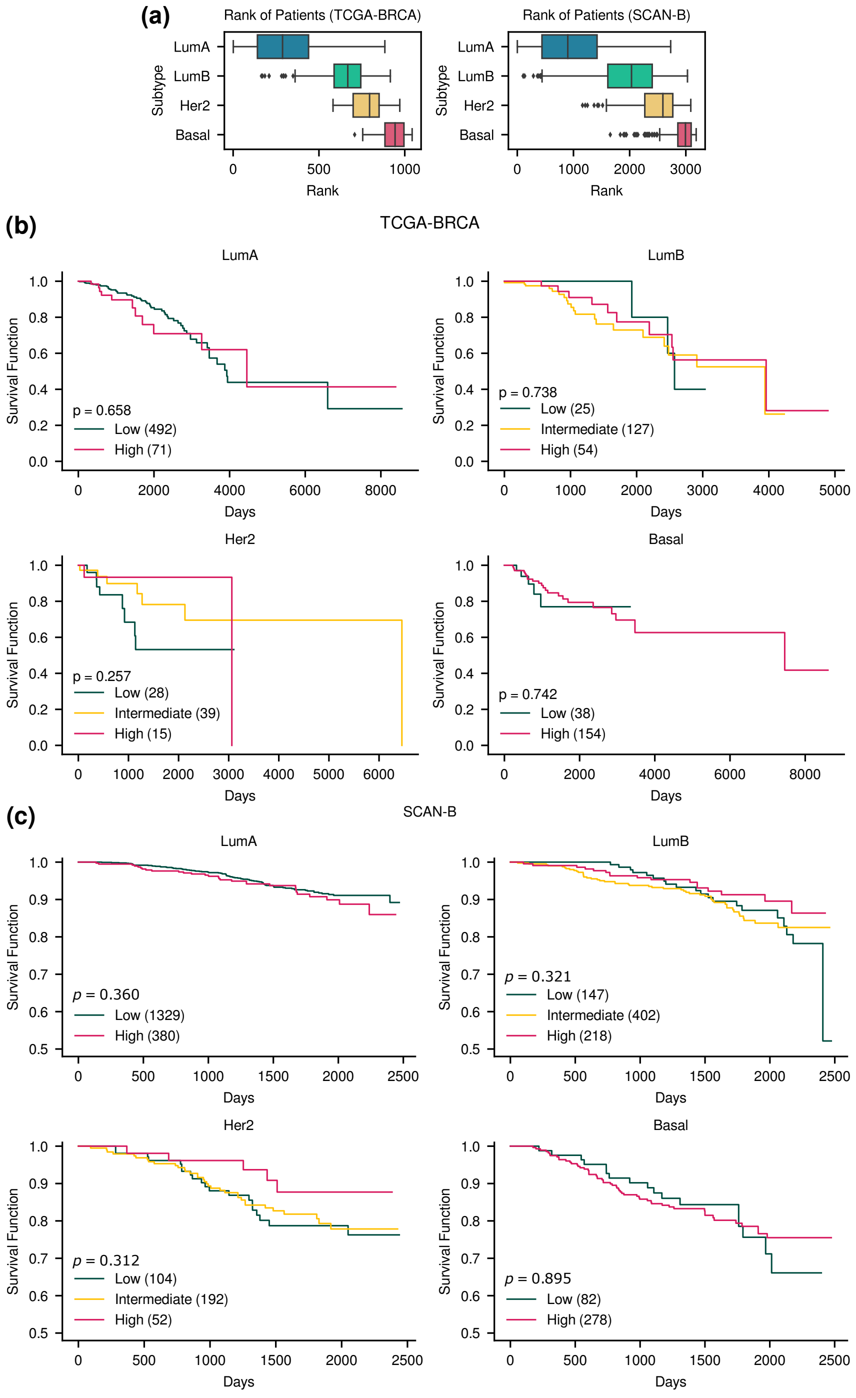

Figure S5: The results when only stratification score was considered ($\lambda = 0$). **(a)** Ranking of patients determined from the final chromosome obtained from the genetic algorithm. **(b,c)** Kaplan-Meier curves for predicted risk groups within each subtype. Significant differences were not observed among groups in the order of risk. The number in parentheses means the number of samples. P-values were results of multivariate log-rank tests. **(b)** TCGA-BRCA, **(c)** SCAN-B.

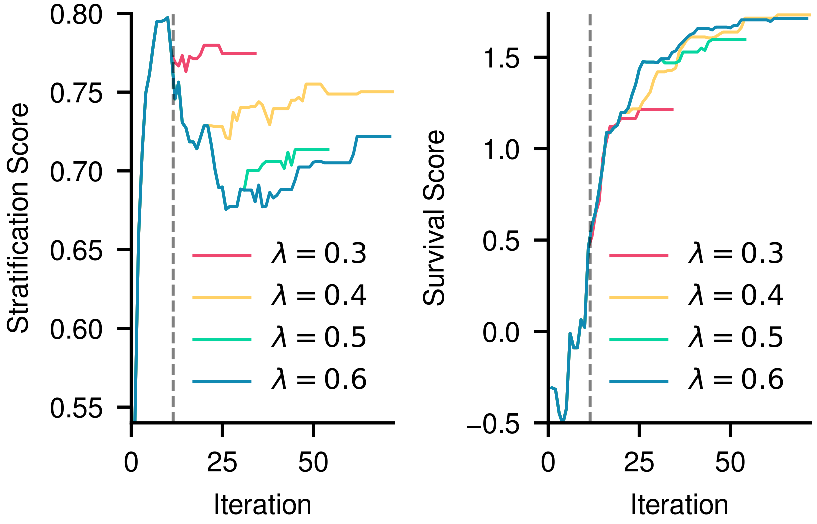

Figure S6: The scores according to the change in λ value. It was observed that lower lambda values tend to focus more on subtype stratification. The black dashed line stands for the point at which the survival score was calculated. Although the survival score was not initially used to evaluate the order of patients, the values were computed and displayed.

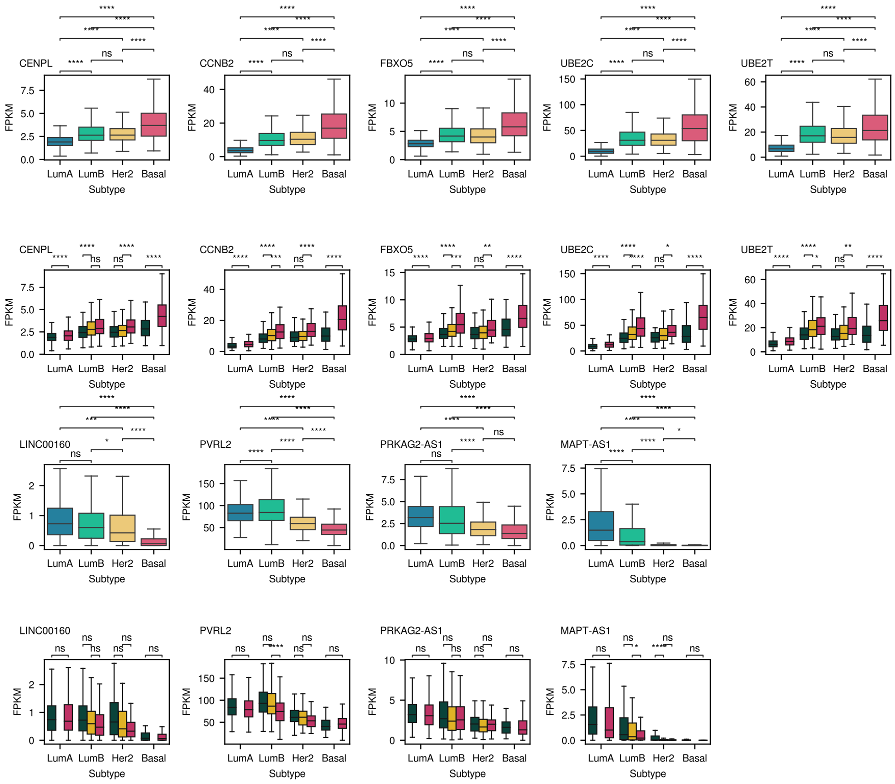

Figure S7: The gene expression levels of frequently selected genes for each subtype and the risk groups predicted within each subtype in SCAN-B data. *CENPL*, *CCNB2*, *FBXO5*, *UBE2C*, and *UBE2T* were selected as a gene related to poor prognosis when its expression level is high. *LINC00160*, *PVRL2*, *PRKAG2-AS1*, and *MAPT-AS1* were selected as a gene associated with poor prognosis when its expression is low. The p-values are the results of t-test with Bonferroni correction. Outliers were omitted. (ns: non-significant, *: $p < 0.05$, **: $p < 0.01$, ***: $p < 0.001$, ****: $p < 0.0001$)

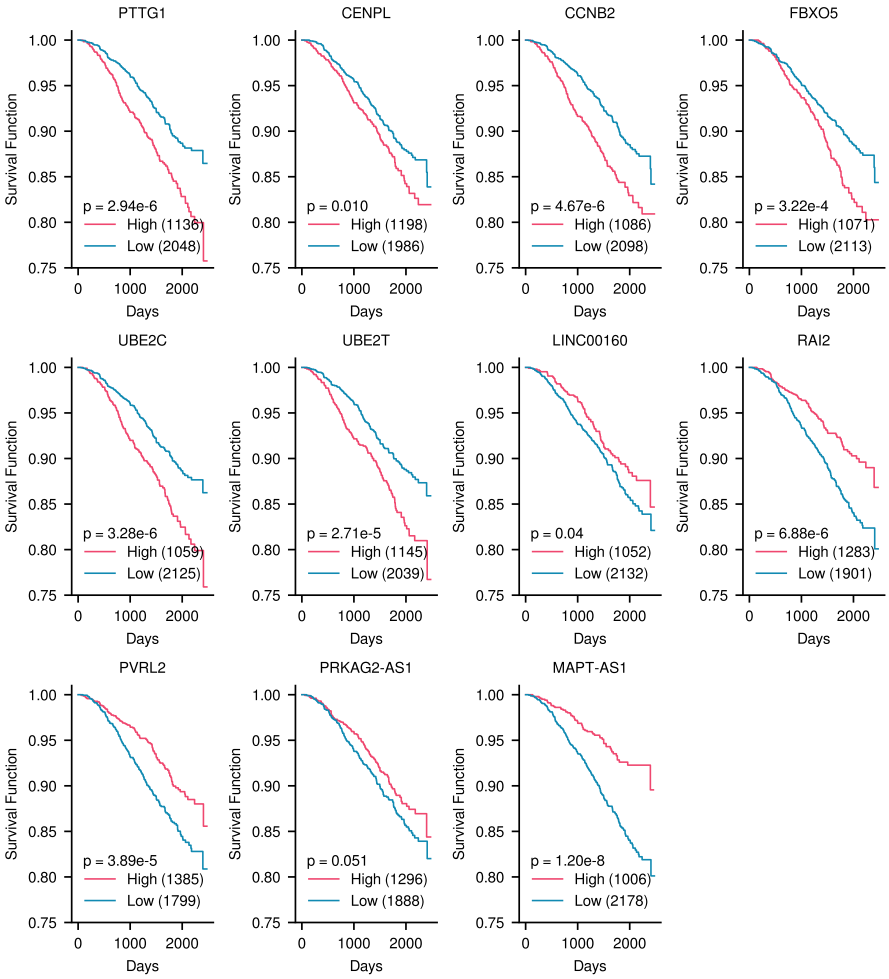

Figure S8: Kaplan-Meier curves of frequently selected genes for SCAN-B data. The samples were split into two groups based on average expression values. The number in parentheses means the number of samples. P-values were results of log-rank tests.

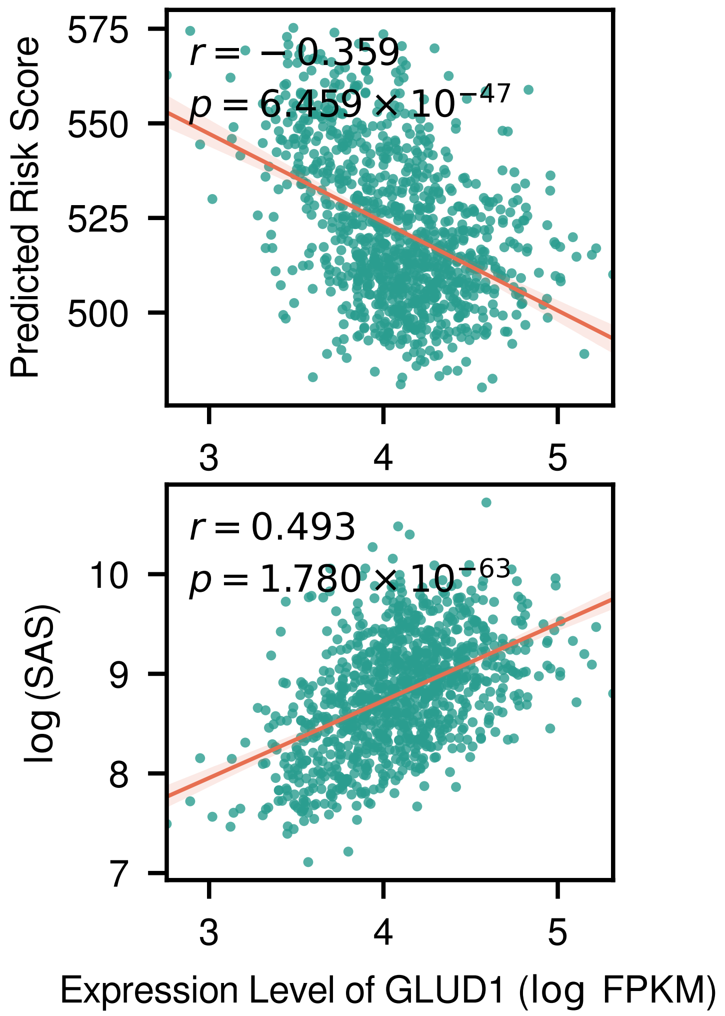

Figure S9: The expression level of *GLUD1* showed a negative correlation with the risk score and a positive correlation with the activity of nitrogen metabolism.
